# Strontium-doped apatitic bone cements with tunable antibacterial and antibiofilm ability

**DOI:** 10.3389/fbioe.2022.969641

**Published:** 2022-12-09

**Authors:** Massimiliano Dapporto, Marta Tavoni, Elisa Restivo, Francesca Carella, Giovanna Bruni, Laura Mercatali, Livia Visai, Anna Tampieri, Michele Iafisco, Simone Sprio

**Affiliations:** ^1^ Institute of Science, Technology and Sustainability for Ceramics (ISSMC) (Former ISTEC), National Research Council (CNR), Faenza, Italy; ^2^ Molecular Medicine Department, Center for Health Technologies, UdR INSTM, University of Pavia, Pavia, Italy; ^3^ Department of Chemistry, Physical Chemistry Section, Center for Colloid and Surfaces Science, University of Pavia, Pavia, Italy; ^4^ Osteoncology Unit, Bioscience Laboratory, IRCCS Istituto Romagnolo per lo Studio dei Tumori (IRST) “Dino Amadori”, Meldola, Italy; ^5^ Medicina Clinica-Specialistica, UOR5 Laboratorio di Nanotecnologie, ICS Maugeri. IRCCS, Pavia, Italy

**Keywords:** bone cements, hydroxyapatite, drug delivery, tetracycline, bone regeneration, antibacterial, antibiofilm

## Abstract

Injectable calcium phosphate cements (CPCs) represent promising candidates for the regeneration of complex-shape bone defects, thanks to self-hardening ability, bioactive composition and nanostructure offering high specific surface area for cell attachment and conduction. Such features make CPCs also interesting for functionalization with various biomolecules, towards the generation of multifunctional devices with enhanced therapeutic ability. In particular, strontium-doped CPCs have been studied in the last years due to the intrinsic antiosteoporotic character of strontium. In this work, a SrCPC previously reported as osteointegrative and capable to modulate the fate of bone cells was enriched with hydroxyapatite nanoparticles (HA-NPs) functionalized with tetracycline (TC) to provide antibacterial activity. We found that HA-NPs functionalized with TC (NP-TC) can act as modulator of the drug release profile when embedded in SrCPCs, thus providing a sustained and tunable TC release. *In vitro* microbiological tests on *Escherichia coli* and *Staphylococcus aureus* strains proved effective bacteriostatic and bactericidal properties, especially for the NP-TC loaded SrCPC formulations. Overall, our results indicate that the addition of NP-TC on CPC acted as effective modulator towards a tunable drug release control in the treatment of bone infections or cancers.

## 1 Introduction

The development of scaffolds for the regeneration of bone defects has been a major research target in the orthopedic field in the last decades, especially given the steady rise of traumas or metabolic diseases such as osteoporosis and bone tumors (Sohn and Oh, 2019; Reid, 2020; Zhao et al., 2021). Biomaterials based on calcium phosphates and hydroxyapatite (HA), are widely considered as elective for the development of bone scaffolds, due to their high chemical similarity with the mineral phase of bone tissue, which is a key aspect to promote new bone formation (Jeong et al., 2019; Lodoso-Torrecilla et al., 2021; Tavoni et al., 2021). However, one of the major issue related to the implantation of scaffolds for bone regeneration refers to post-operative infections, in particular to the formation of bacterial biofilms on the surfaces of the implants which usually leads to the failure of the implant (Li and Webster, 2018).

Hence, the development of bioactive bone substitutes combining effective regenerative and antibacterial features over time is highly demanded (Huang and Brazel, 2001; Johnson and Garcia, 2015; Ferracini et al., 2018).

In this context, a promising perspective is given by calcium phosphate bone cements (CPCs) (Ginebra et al., 2012; Xu et al., 2017; Yousefi, 2019). Their preparation involves the mixing of a powder and a liquid, thus obtaining injectable pastes, potentially suitable for mini-invasive implantation procedures in bony defects with complex geometry, such as the spine, the femur head, the tibial plateau (Ginebra et al., 2012; Magnan et al., 2013; Yousefi, 2019). Their ability of self-hardening by chemical reactions acting at body temperature allows the tailoring of physico-chemical and structural features such as chemical composition, ion doping and nanostructure, which are relevant for the bioactivity and inherent antibacterial properties (Wu et al., 2018; Sprio et al., 2019; Sprio et al., 2020), as well as to achieve effective mechanical properties (Brown and Chow, 1983; Ginebra et al., 2012). An interesting feature of CPCs is also related to their higher specific surface area and micro-porosity (Tampieri et al., 2019), both potential key aspects for the linking and controlled release of various bioactive molecules, particularly if compared with sintered apatitic scaffolds (Grossin et al., 2010; Farbod et al., 2016).

The development of drug-loaded bone cements has been previously studied, with the prevalent approach to mix the drug, in dry powder state, with the solid component of cements (Liu et al., 2010; Bistolfi et al., 2011; Mori et al., 2011; Qin et al., 2015; Cara et al., 2020). However, the development of drug loaded self-hardening apatitic formulations with appropriate injectability, flowability and setting times is widely recognized as a difficult task (Ginebra et al., 2010; Wang et al., 2014; Zhang et al., 2014; Pastorino et al., 2015; O'Neill et al., 2017; Anagnostakos et al., 2022; Ghosh et al., 2022; Hu et al., 2022). For instance, the loading of antibiotic tetracycline (TC) from CPCs was associated to a delaying effect on the hardening and on the rate of formation of the final HA phase (Ratier et al., 2004; Rabiee, 2013). This was attributed to the chemical affinity of TC with Ca^2+^ ions, which could interfere with the dissolution-reprecipitation process responsible of the transformation of the CaP precursors into HA and of the cement setting times (Akashi et al., 2001; Ratier et al., 2001; Tamimi et al., 2008).

In the last decades, Sr-doped CPCs gained increasing interest due to antiosteoporotic and osteointegrative character (Schumacher and Gelinsky, 2015; Lode et al., 2018; Sun et al., 2021). In this work, we synthesized a self-hardening paste based on strontium-substituted HA by using Sr-doped α-tricalcium phosphate powders (α-(Sr,Ca)_3_(PO_4_)_2_: Sr-αTCP) as unique solid precursor (Sprio et al., 2016; Dapporto et al., 2021). We prepared a Sr-αTCP precursor powder designed with Sr/(Sr + Ca) = 2 mol%, as suggested by our previous study where such a small amount of Sr^2+^ ions doping resulted effective in enhancing stem cell proliferation and modulating osteoblast and osteoclast cells fate *in vitro*, targeting the re-equilibration of the natural bone turnover in osteoporosis scenario (Montesi et al., 2017). Before mixing with the liquid component, the precursor powder was added with TC-loaded HA nanoparticles (HA-NPs), appositely synthesized to achieve a modulation of TC release. The TC was selected as a broad-spectrum antibiotic capable to contrast post-operative infections. The incorporation of unfunctionalized HA-NPs into CPCs has been previously investigated, resulting into higher dynamic viscosity and final compression strength of the cement (Montufar et al., 2013).

Herein, we first optimized the TC adsorption process on the HA-NPs and then the TC release profiles from SrCPC functionalized with TC-loaded HA-NPs were investigated in physiological conditions. Furthermore, we assessed the effect of the functionalization with TC on the overall microstructure and mechanical properties of cement.

To assess the antibacterial activity of TC-loaded CPCs, two reference infective bacterial strains, i.e.,: *Escherichia coli* and *Staphylococcus aureus* were used. In addition, TC-loaded CPCs were tested against bacterial biofilms to evaluate its capability to inhibit biofilm formation and/or disrupting preformed biofilms. Physico-chemical mechanisms inherent to the chemical composition and multiscale structure of the hardened bone substitute, potentially affecting the drug release profile and the antibacterial properties, were also investigated, and discussed.

## 2 Materials and methods

### 2.1 Preparation of Sr-doped calcium phosphate cements

The inorganic precursor powder and liquid components of the SrCPC cement were prepared according to a previous work (Sprio et al., 2016). Briefly, a Sr-doped α-tricalcium phosphate solid precursor (Sr-αTCP) was prepared by mixing calcium carbonate (CaCO_3_, Sigma Aldrich, St. Louis, MO, United States), dicalcium phosphate dibasic anhydrous (CaHPO_4_, Sigma Aldrich) and strontium carbonate (SrCO_3_, Sigma Aldrich), followed by thermal treatment at 1400°C for 1 h and rapid cooling, to obtain a final composition of Sr/(Ca + Sr) ≈ 2 mol% (hereinafter coded as SrCPC). Such a powder was milled by planetary mono mill (Pulverisette 6 classic line, Fritsch, Germany) for 50 min at 400 rpm using a zirconia jar with 5 mm diameter grinding media. The liquid component of the paste was made of aqueous solutions of disodium hydrogen phosphate dihydrate, 5 wt% (Na_2_HPO_4_∙2H_2_O, Fluka) and sodium alginate, 2 wt% (Alginic Acid Sodium Salt from Brown Algae, Sigma Aldrich). Finally, appropriate amounts of powder and liquid, according to liquid-to-powder (LP) ratio equal to 0.6, were mixed to obtain the SrCPC cements.

### 2.2 Synthesis of hydroxyapatite nanoparticles

Hydroxyapatite nanoparticles (HA-NPs) were synthesized as previously reported (Sandhofer et al., 2015). Briefly, a solution of H_3_PO_4_ (0.21 M) was dropped into a solution of Ca(CH_3_COO)_2_ (0.35 M) maintaining pH = 10 by addition of NH_4_OH. This mixture was stirred at room temperature overnight, then followed by powder sedimentation (2 h) and washing with ultrapure water by multiple centrifuges at 5000 rpm. Finally, HA-NPs were freeze-dried under vacuum (3 mbar) overnight.

### 2.3 Functionalization of NPs with tetracycline

Firstly, a calibration curve of TC (C_22_H_25_ClN_2_O_8_, Sigma Aldrich) was investigated in 4-(2-hydroxyethyl)-1-piperazineethanesulfonic acid (HEPES) buffer solutions (0.01M, with KCl 0.01M, pH 7.4), for concentrations in the range 0.005–2 mg·ml^−1^, in dark conditions to prevent the photo-degradability of the drug when exposed to UV radiation (Gomez-Pacheco et al., 2012). The resulted line (*R* ^2^ = 0.999) was obtained by monitoring the optical density at 355 nm by UV-vis spectrophotometry (LAMBDA™ 750 UV/Vis/NIR spectrophotometer from PerkinElmer), considering a molar adsorption coefficient of TC of ε = 136 L mol^−1^∙cm^−1^. Then, the adsorption kinetic of TC on HA-NPs was investigated by dispersing 20 mg of HA-NPs into 5 ml of TC solutions (1 mg/ml), in a thermostatic stirrer at 37°C up to 24 h. The supernatant was collected at several timepoints after centrifuging the suspensions at 12,000 rpm for 2 min, then analyzed by UV spectroscopy and completely refreshed. In this way, the adequate incubation time to maximize the TC adsorption on HA-NPs was found. Then, the adsorption of increasing amount of TC was also explored (0.1 and 1 mg/ml), with the same solid/liquid ratio and temperature used to determine the adsorption kinetic. According to this protocol, the optimal incubation time and TC concentration to optimize the drug loading were defined. As control samples, TC-free NPs solutions were also prepared and analyzed.

### 2.4 Preparation of TC-loaded calcium phosphate cements (SrCPC_NP-TC)

Preliminary experiments were carried out to evaluate the effect of the dry addition of increasing amounts of NPs, in the range 0–15 wt% in respect to the Sr-αTCP precursor, on the setting times and injectability of the SrCPC. In this way, the maximum amount of NPs capable to avoid significant variations of SrCPC setting times and viscosity was identified. Then, the same amount of HA-NPs functionalized with TC (hereafter code as NP-TC) was dry mixed with SrCPC in different amounts, before mixing with the liquid component, to prepare NP-TC loaded cements (hereinafter coded as SrCPC_NP-TC). The powder and liquid components were mixed using a high-energy planetary shear-mixer (Thinky Mixer ARE-500, Thinky, Japan) at 1000 rpm for 90 s. The effect of NP addition was tested by preparing also control TC-loaded SrCPC samples without NP-TC (hereinafter coded as SrCPC_TC), by dry mixing TC to the Sr-αTCP precursor. In this latter sample the total amount of TC was the same of that included in the SrCPC_NP-TC. In this case, the presence of TC did not affect the rheological performance of cements.

### 2.5 Physico-chemical characterizations

The crystallographic features of the samples were investigated by X-ray diffraction (XRD) on a D8 Advance diffractometer (Bruker, Karlsruhe, Germany), with CuKα radiation, 2θ range 10–80, scan step 0.02). The amount of αTCP and HA crystalline phases was quantified by JCPDS file 029-0359 and 009-0432, respectively. The average crystal size was calculated by Scherrer’s formula D = k∙λ/(B∙cosθ), where D is the crystalline diameter, k is the shape constant (≈0.9), λ is the radiation wavelength ≈1.5406 Å, θ is the Bragg angle, and B is the full width at half-maximum of the observed peak.

The specific surface area (SSA) was calculated using the Brunauer−Emmett−Teller (BET) method (Surfer, Thermo Scientific, United States), from the nitrogen adsorption data at a relative pressure of 0.03 Torr; the measurement error is related to the accuracy of N_2_ adsorption/desorption techniques (<1%). Fourier-transform infrared spectroscopy with attenuated total reflection (FTIR-ATR) (Nicolet iS5, Thermo Scientific) was investigated in the range 400–4000 cm^−1^. Dynamic light scattering (DLS) (Malvern, Zetasizer Nano ZSP) was performed to investigate the zeta potential of the samples by dispersing NPs (1 mg/ml) in a buffer solution at pH 7.4. The initial and final setting times of the cement formulations were monitored by Gillmore needles, according to standard ASTM C266-99.

The morphology of SrCPC scaffolds was explored by Scanning Electron Microscopy (SEM), using a Zeiss EVO-MA10 scanning electron microscope (Carl Zeiss, Oberkochen, Germany) at 20 kV acceleration voltage.

Energy Dispersive X-Ray Spectrometry (Oxford Scientific, OXFORD INCA Energy 350 X-Max 50, Oxford, United Kingdom) associated with Scanning Electron Microscopy (SEM) (Zeiss, EVO MA10-HR “dual gun”, Oberkochen, Germany) at 20 kV, was used to investigate the percentage of the elements (Ca, P, Sr) present on the surface of SrCPC**.**

The compressive strength of cements was evaluated by testing cylindrical specimens (n. Five samples; diameter = 8 mm; height = 17 mm) obtained after hardening in Teflon moulds for 30 min and then immersed in HEPES solution at 37°C for 7 days. The tests were performed in displacement control at 1 mm/min by a universal testing machine (Zwick Roell Z050).

### 2.6 Bacterial strains and bacterial biofilms culture conditions

The microorganisms used were a Gram-negative strain, *Escherichia coli* ATCC 25922 (*E. coli*) and a Gram-positive strain, *Staphylococcus aureus* ATCC 25923 (*S. aureus*) (Kesici et al., 2019; Trespidi et al., 2021), kindly obtained from the laboratory of Prof. R. Migliavacca (Department of Clinical-Surgical Diagnostic and Pediatric Sciences, Unit of Microbiology and Clinical Microbiology, University of Pavia, Italy). Bacteria were grown in 10 ml of appropriate medium, overnight, under aerobic conditions at 37°C using a shaker incubator (VDRL Stirrer 711/CT, Asal Srl, Italy). *E. coli* was inoculated in Luria Bertani broth (LB) (ForMedium™, UK) whereas *S. aureus* in TSB (Tryptic Soy Broth) (ForMedium™, UK).

The number of bacterial cells/ml of both cultures was determined by comparing the optical density (OD600) of the sample with a standard curve relating OD600 to cell number (Bari et al., 2017; Sprio et al., 2020; Villani et al., 2020).

### 2.7 Evaluation of antibacterial and antibiofilm activity of scaffolds

The antimicrobial activity of SrCPC-TC and SrCPC_NP-TC formulation was tested on cylindrical specimens (diameter = 10 mm; height = 3 mm), previously sterilized with a dose of 25 kGy gamma rays. No reduction in antibiotic release capacity of sterilized samples was observed in comparison with unsterilized ions-doped apatitic bone cements (data not shown). The antimicrobial activity was performed on all the cylindrical specimens with both bacterial strains.

The viability was estimated through the quantitative 3-(4,5-dimethylthiazol-2-yl)-2,5-diphenyltetrazolium bromide (MTT) colorimetric assay (Sigma-Aldrich, St. Louis, SM, United States). This test measures dehydrogenase activity as an indicator of the bacterial metabolic state. MTT solution (5 mg/ml), dissolved in sterile PBS (0.134 M NaCl, 20 mM Na_2_HPO_4_, 20 mM NaH_2_PO_4_), was used as a stock solution and the working concentration was 0.5 mg/ml. The test was performed for 3 h at 37°C. Upon presence of viable bacteria, reduction of the MTT salt results in purple insoluble formazan granules that are dissolved in acidified 2-propanol (0.04 N HCl). The colorimetric reaction was analyzed at CLARIOstar (BMG Labtech, Ortenberg, Germany) at 570 nm wavelength with 630 nm as reference wavelength. Results were normalized to bacterial cells cultured with LB medium of Tissue Culture Plate (TCP = Control). All the viability experiments were carried out in triplicate and repeated 2 times.

#### 2.7.1 Direct and indirect contact experiments with planktonic bacteria

Sterile scaffolds were washed twice in sterile ddH_2_O. Two types of assays were performed in planktonic conditions: direct and indirect contact.

##### 2.7.1.1 Direct contact experiment

600 µl of 1*10^4^ of each bacterial strain were incubated for 6 h, 24 h and 48 h, at 37°C, onto the cements, and in tissue culture plate (Ctrl). The viability has been assessed either onthe supernatant of planktonic bacteria kindly removed from the scaffolds (analysis 1); or on the bacteria adherent onto the scaffold’s surface (analysis 2). In particular:

**Analysis 1)**: 600 µl of the bacterial supernatants were added with 60 µl of MTT and incubated for 3 h at 37°C. **Analysis 2)**: After removal of planktonic bacteria the SrCPC scaffolds were washed twice in PBS 1X and transferred into a 15 ml tube with 600 µl of PBS 1X. The tubes were vortexed to allow the detachment of bacteria. Aliquots of 100 µl were transferred into a 96-well plate and the viability was determined as previously described. Furthermore, after this treatment, the scaffolds were incubated on agar plates to confirm the absence of live bacterial cells for each strain.

##### 2.7.1.2 Indirect contact experiment

LB (0.5 ml) medium was incubated with each scaffold placed at the bottom of a 24-well sterile culture plate (Euroclone S. p.A, Italy), overnight at 37°C to allow release of ions and tetracycline. No pH changes in LB medium were observed.

Two-fold serial dilutions of the overnight solutions were performed starting from a volume of 100 µl of solution. 100 µl of 1*10^4^ bacteria were inoculated and incubated for 24 h at 37°C. The viability was determined by MTT assay as previously described.

#### 2.7.2 Anti-biofilm experiments

Sterile scaffolds were washed twice in sterile ddH_2_O. The assays were performed for both bacterial strains in two types of conditions: pre-biofilm and post-biofilm conditions.

##### 2.7.2.1 Pre-biofilm culture conditions

Overnight cultures of *E. coli* and *S. aureus* were diluted to 1*10^7^/sample in LB containing 0.5% glucose for *E. coli* and 0.25% for *S. aureus* (Pallavicini et al., 2017). Aliquots of 600 µl of the diluted bacterial suspensions were directly seeded onto the scaffolds contained in a 24-well culture plates (Euroclone S. p.a, Italy), and incubated for 24 h at 37°C. After the incubation time, the scaffolds were washed twice with PBS 1X and transferred into a 15 ml tube with 600 µl of PBS 1X. The tubes were vortexed to allow the detachment and resuspension of biofilm. Aliquots of 100 µl were transferred into a 96-well plate to evaluate cell viability with MTT assay as previously described (Section 2.7).

##### 2.7.2.2 Post-biofilm culture conditions

Firstly, the TC and ions release from the nude scaffolds in LB medium were performed in sterile conditions as previously described (indirect contact experiment). Afterwards, to allow the formation of a biofilm of both bacterial strains, 200 µl of 1*10^7^ bacteria (cultured overnight) were diluted in glucose-containing LB, directly plated in 96-well flat-bottomed sterile polystyrene microplates (Euroclone S. p.a, Italy) and incubated for 24 h at 37°C. After overnight incubation, the supernatant, containing planktonic bacteria, was carefully removed from the preformed bacterial biofilms. Two-fold serial dilutions of LB medium containing the released TC and ions from the nude scaffolds were added to the preformed biofilms for 24 h at 37°C. After the incubation time, each biofilm was washed twice with sterile PBS 1X and resuspended to detect cell viability by MTT assay as previously described (Section 2.7).

### 2.8 Scanning electron microscopy (SEM) of scaffolds with bacterial biofilms

Bacteria were diluted as previously described in Section 2.7.2. After 24 h of incubation at 37°C, in pre- and post-biofilm conditions, the planktonic bacteria were removed and the biofilms were washed carefully with PBS 1X and fixed with 2.5% (v/v) glutaraldehyde (Sigma-Aldrich, St. Louis, SM, United States) in 0.1 M Na-cacodylate buffer (Sigma-Aldrich, St. Louis, SM, United States) (pH 7.2), for 1 h at 4°C. After two washes with Na-cacodylate, to remove excess of glutaraldehyde, *E. coli* biofilms were dehydrated just with two washes of 96% ethanol (Merck Life Science S. r.l, Milano, Italy) for 10 min whereas *S. aureus* biofilms were dehydrated using increasing concentrations of ethanol (25, 50%, 75%) for 5 min and two washes of 96% ethanol for 10 min. The samples were lyophilized for 3 h using a K-850 apparatus (Emitech Ltd., Ashford, UK) and placed on a mounting base. Finally, they were gold sputtered and images were acquired using a Zeiss EVO-MA10 scanning electron microscope (Carl Zeiss, Oberkochen, Germany), 20 kV acceleration voltage.

### 2.9 Statistical analysis of microbiological tests

All the statistical calculations were carried out by considering the mean of the results (in triplicate) obtained from two separate experiments. The analysis was carried out using GraphPad Prism 9 (GraphPad Inc, San Diego, CA, United States). Statistical analysis was performed using Student’s unpaired, two-sided *t*-test (significance level of *p* < 0.05). In addition, two-way analysis of variance (ANOVA), followed by Bonferroni’s multiple comparisons test was performed.

## 3 Results and discussion

### 3.1 Physico-chemical features of HA-NPs

**FIGURE 1:**
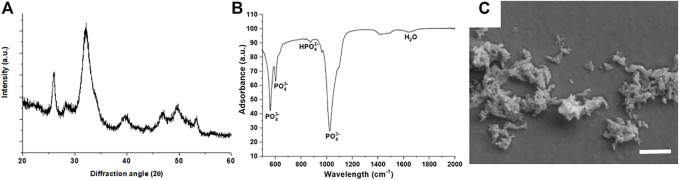
**(A)** X-ray diffraction pattern of HA-NPs; **(B)** FTIR-ATR spectrum of HA-NPs; **(C)** SEM micrograph of HA-NPs (scale bar = 500 nm).

The XRD analysis (Figure 1A) of HA-NPs reveals the typical pattern of HA (hexagonal, space group P6_3_/m, JCPDS file 09–0432), with marked peak broadening related to the small crystal size according to the synthesis temperature (T = 40°C) (Sprio et al., 2019). No other crystalline phases were detected.

The average crystalline domain size, evaluated by the Scherrer’s formula, was 13.4 ± 2.4 nm, thus confirming the nano-crystallinity of HA-NPs. The SSA of the HA-NPs was 160.05 m^2^/g. The FTIR-ATR spectrum (Figure 1B) confirms the vibrational signatures of HA, particularly all vibration modes of PO_4_^3-^, including the characteristic bands for ν_1_, ν_2_, ν_3_ and ν_4_ stretching modes at 963, 472, 1040 and 560–600 cm^−1^, respectively, were detected (Ahmed et al., 2015). SEM analysis (Figure 1C) confirms the nano-size and the needle-like morphology of HA-NPs, which is consistent with the high SSA value found by BET method.

### 3.2 Investigation of addition of HA-NPs on SrCPC properties

**FIGURE 2:**
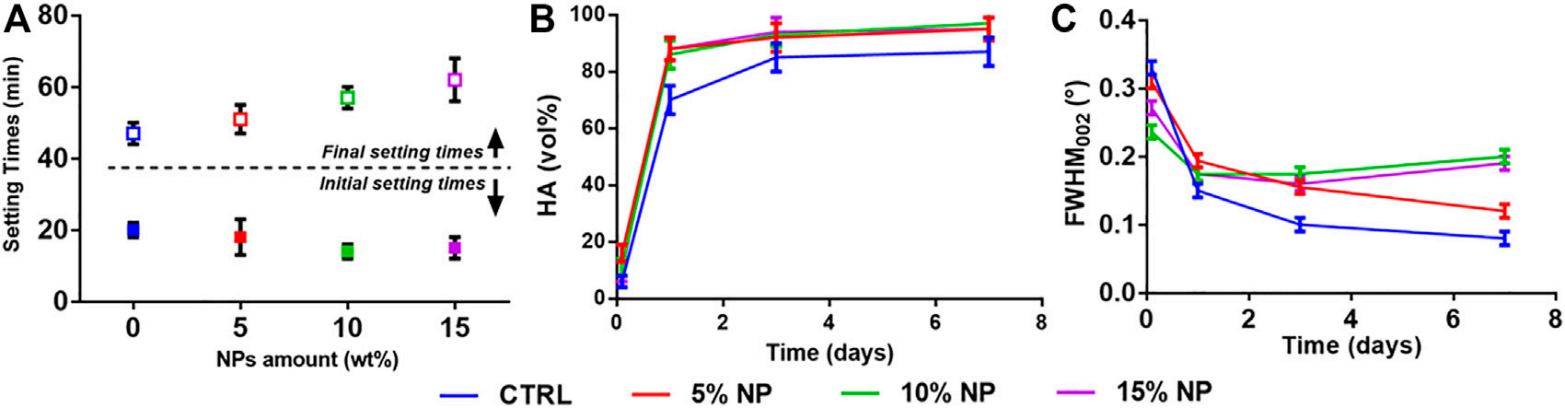
Effect of HA-NPs on SrCPC properties: **(A)** Initial and final setting times; **(B)** Phase transformation into HA up to 7 days; **(C)** Full-Width at Half Maximum (FWHM) of the (002) peak up to 7 days.

A preliminary set of experiments was carried out to determine the maximum extent of HA-NPs that could be added to SrCPCs without significant variations in setting times and injectability, keeping into account that an initial setting time of ∼15–20 min is considered as suitable to meet the clinical practice requirements (Driessens et al., 1998; Bohner, 2007). We observed a slight decrease of the initial setting times, associated with a marked increase of the final setting time, when raising the concentration of HA-NPs in the cement (Figure 2A).

After the initial setting, the cements were analyzed by XRD along 7 days, to quantify the extent of transformation into HA. We detected enhanced HA formation in the cements enriched with HA-NPs (i.e., in the range 10–20 vol% more than the control, see Figure 2B), ascribable to the ability of HA-NPs to act as seeds for heterogeneous nucleation of HA (Driessens et al., 1993; Ginebra et al., 2012; Montufar et al., 2013), thus enhancing the dissolution/reprecipitation process. Interestingly, we detected an increasingly lower crystallinity of the HA phase forming the cement in samples containing HA-NPs, as attested by the increase of Full Width at Half Maximum (FWHM) of the HA (002) reflection (Figure 2C), which is inversely related to the crystalline domain size along the *c* axis of the HA lattice (Pleshko et al., 1991; Poralan et al., 2015). This finding suggests that the presence of HA-NPs, besides favoring the cement setting and phase transformation process, also represents a hindering factor for the crystal growth of the new HA phase, which can be beneficial to achieve higher bioactivity. The addition of 10wt% HA-NPs, in respect to Sr-αTCP amount, was finally selected as optimal amount for the preparation of CPCs formulations with performance compliant for clinical applications.

### 3.3 Investigation of TC adsorption on NPs and release

**FIGURE 3:**
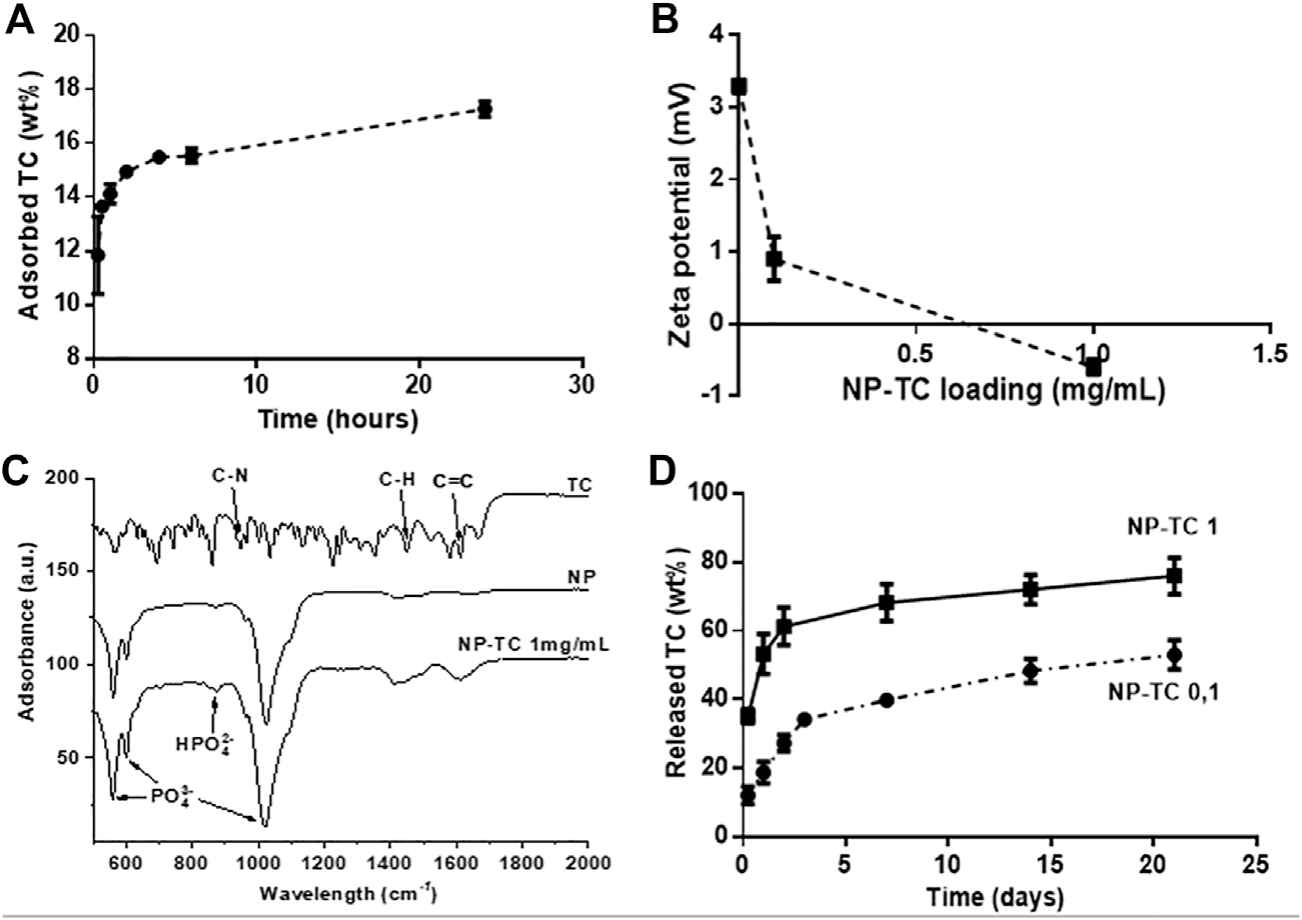
**(A)** TC adsorption kinetic on HA-NPs (TC concentration = 1 mg/ml); **(B)** Zeta Potential analysis of NP-TC; **(C)** FTIR-ATR spectra for TC, NP and NP-TC 1 mg/ml; **(D)** Release profile of TC from NPs, functionalized with both 0.1 and 1 mg/mL TC concentration.

Experiments were carried out to optimize the adsorption of TC on HA-NPs (NP-TC). The adsorption kinetic of a TC solution on HA-NPs at incubation times up to 24 h evidenced the time of 6 h as adequate soaking time to achieve a quasi-equilibrium condition (Figure 3A **)**.

Then, given the optimal contact time of 6 h, the adsorption was evaluated at different TC model concentrations (Figure 3B). The adsorbed amount of TC on HA-NPs increased as a function of TC in solution, reaching a quantity of about 880.8 μg/m^2^. Pristine HA-NPs exhibited slightly positive zeta potential, while a decrease in zeta potential was observed with increasing the TC concentration. In this respect, TC was reported as a amphoteric molecule with ionizable groups (i.e., a tricarbonylamide group, a phenolic diketone group, and a dimethyl amino group) capable to undergo protonation or deprotonation reactions as a function of pH (Chang et al., 2015). A pH-sensitive behavior of TC was previously observed, in particular three different forms exist in function of pH: *cationic*, at pH < 3.3, *zwitterionic*, at pH 3.3–7.7, and *anionic* at pH > 7.7; when pH reaches above 7.0, about 25% of TC exists in the anionic form (Gopal et al., 2020). The rise of negative surface with increasing the TC amount confirms such behavior as the pH was kept at 7.4 namely in the reported range for TC zwitterionic form, but also closely borderline with anionic character of TC.

The loading of TC on NPs was monitored by FTIR-ATR analysis (Figure 3C), highlighting vibrational peaks of TC at 1648–1582 cm^−1^ assigned to C=C stretching, aromatic C-H bending at 1458 cm^−1^ and CH_3_ bending at 1357 cm^−1^ (Trivedi et al., 2015; Qayoom et al., 2020). Aromatic in-plane and out-plane deformation peaks were detected in the range 1247–1000 cm^−1^ and 567–501 cm^−1^, respectively, whereas the vibrational peak at 965 cm^−1^ was assigned to C-N stretching. The adsorption of TC on the surface of HA-NPs is attested especially by the increased intensity of the adsorption bands at 1640 and 1420 cm^−1^, assigned to C=C stretching and aromatic C-H bending, respectively. Figure 3D shows that the TC release profiles from differently loaded HA-NPs feature similar trend, reaching a plateau after 7 days. The sample loaded with 1 mg/ml TC solution (NP-TC1) exhibited increased amount of released TC along the first 3 days and also after 21 days (∼80 wt%), in comparison with the sample loaded with 0.1 mg/ml (NP-TC 0.1). Such long-term release profile suggested a stable linking of TC to the surface of HA-NPs, possibly facilitated by the presence of charged anionic groups (such as phosphate) on apatite surface (Drouet et al., 2012). In our working conditions the formation of electrostatic interactions between TC and the apatite surface can be hypothesized. The originated ammonium groups of TC are generally associated to low affinity interactions to the surface of apatite. The adsorption of TC molecule on biomimetic apatite powders was previously investigated and modeled (Cazalbou et al., 2015). In this study, the coordination of TC with apatite exhibited negative change in Gibbs free adsorption energy, in a range close to the limit between simple physisorption and chemisorption, ascribed to the absence of very high affinity charged end group on the TC molecules, thus excluding a multilayer adsorption. Another recent computation study also described the bonding between TC and HA as weak coordination interactions including Van der Waals and hydrogen bonds (Song et al., 2022).

### 3.4 Physico-chemical and release properties of NP-TC loaded cements

**FIGURE 4:**
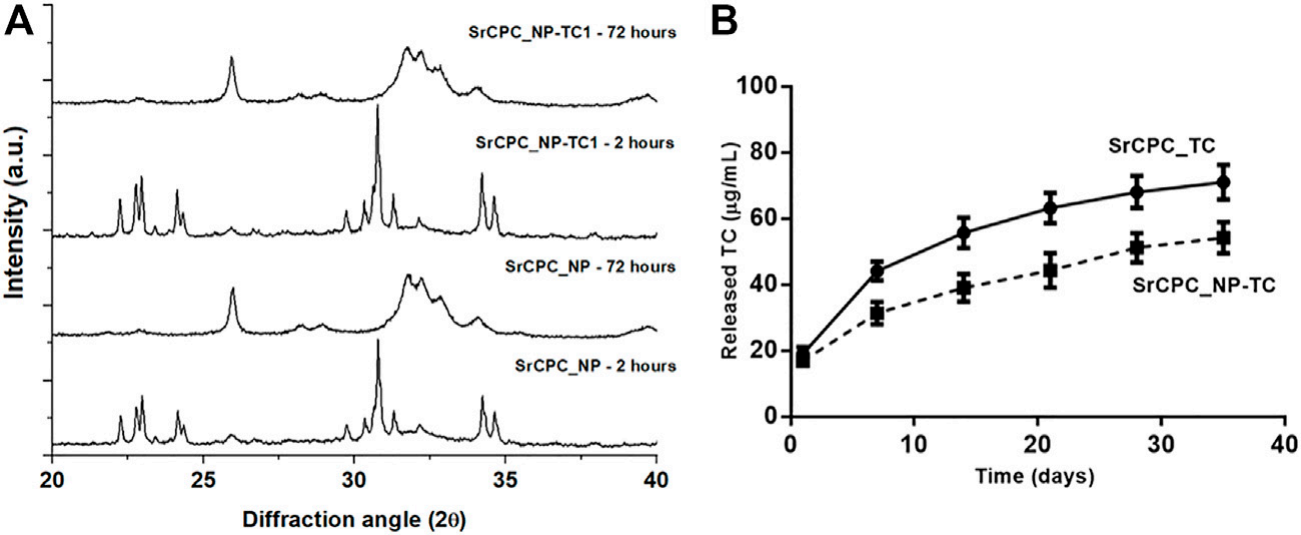
**(A)** XRD of cements with TC (SrCPC_NP-TC1) and without TC (SrCPC_NP) at 2 and 72 h upon mixing and maturation at 37°C; **(B)** Release profile of TC from cements with NPs (SrCPC_NP-TC) and without NPs (SrCPC_TC).

The setting reaction of NP-TC1 loaded SrCPC (SrCPC_NP-TC1) was characterized by XRD (Figure 4A). At 2 h after mixing only αTCP phase was detected, while at 72 h only crystalline HA phase was observed, without any secondary phases. By comparing the XRD patterns of SrCPC_NP-TC1 and SrCPC-NP, we can conclude that the presence of linked TC did not affect the setting process and extent of phase transformation of the precursors into HA.

The effect of NP-TC on the TC release from cements was investigated along 35 days by testing both SrCPC_TC and SrCPC_NP-TC1 cements (Figure 4B). SrCPC_TC cement was obtained by mixing the precursor powder with free TC (i.e., not bound to any HA-NPs) in the same amount detected on NP-TC1 (≈19.2 mg).

Interestingly, despite the same TC amount in both formulations, a significant decrease in TC release was exhibited by the SrCPC_NP-TC1 formulation, for each timepoint. This finding shows that, when linked to HA-NPs, the release profile of TC results further slackened.

The mathematical interpretation of our results was also proposed, according to semiempirical models able to describe drug release from polymeric or monolithic systems, such as Korsmeyer-Peppas or power law model ( $\chi_{i}=Kt^{n}$ ) (Korsmeyer et al., 1983; Fosca et al., 2022), where *χi* is the fraction of the released drug by time t, *K* is a parameter including geometrical and structural features of the matrix, and *n* is as a coefficient related to the mechanism that governs the release kinetic, according to Supplementary Table S1. Table 1 **.** The fitting of empirical data (Figure 3D) is reported in Table 1 and Supplementary Figure S1.

**TABLE 1:** Different values for *n* exponent of Korsmeyer-Peppas model obtained by fitting our empirical data, associated to the corresponding release regime.

Samples	Korsmeyer-Peppas model applied	*n* value	Release regime
NP-TC	$f_{i}=\frac{M_{i}}{M_{\infty }}=Kt^{n}+b$	n = 0.120 ± 0.080	Diffusive regime with hampered release
SrCPC-TC	$f_{i}=\frac{M_{i}}{M_{\infty }}=Kt^{n}$	n = 0.312 ± 0.001	Diffusive regime with hampered release
SrCPC-NP_TC	$f_{i}=\frac{M_{i}}{M_{\infty }}=Kt^{n}$	n = 0.704 ± 0.048	Anomalous transport

The lowest *n* coefficient was calculated for NP-TC sample, exhibiting an initial burst release, thus involving the addition of a constant *b* value into the model, associated to *Diffusive regime with hampered release*. The same Fickian release regime was also associated to SrCPC-TC, on the basis of *n* coefficient. Interestingly, significantly higher *n* coefficient was calculated for SrCPC-NP_TC, confirming that the addition of NP-TC led to a different, non-Fickian, release regime, named *Anomalous transport*. It was hypothesized that the mechanisms underlying this condition basically include a combination of TC diffusion and dissolution of NP-TC or cement matrix.

The rate of degradation of SrCPCs can be considered much lower than the rate of drug release, so that, when the drug was simply mixed with the cement without the use of NPs, the drug release is mainly controlled by the process of diffusion through the cement matrix. It was reported that the presence of the drug into a SrCPC matrix can be generally ascribable to: 1) segregation of the drug in the liquid phase within the micropores of the material, 2) adsorption or chemical bound on the surface of the newly formed crystals, or 3) drug crystals or aggregates, in case of drug concentration higher than the drug solubility in the liquid phase [16]. On this basis, we also hypothesized that the TC is mainly adsorbed on the crystals surface or entrapped, as individual, or aggregated molecules, within the nanopores or micro-voids existing between the entangled HA crystals.

**FIGURE 5:**
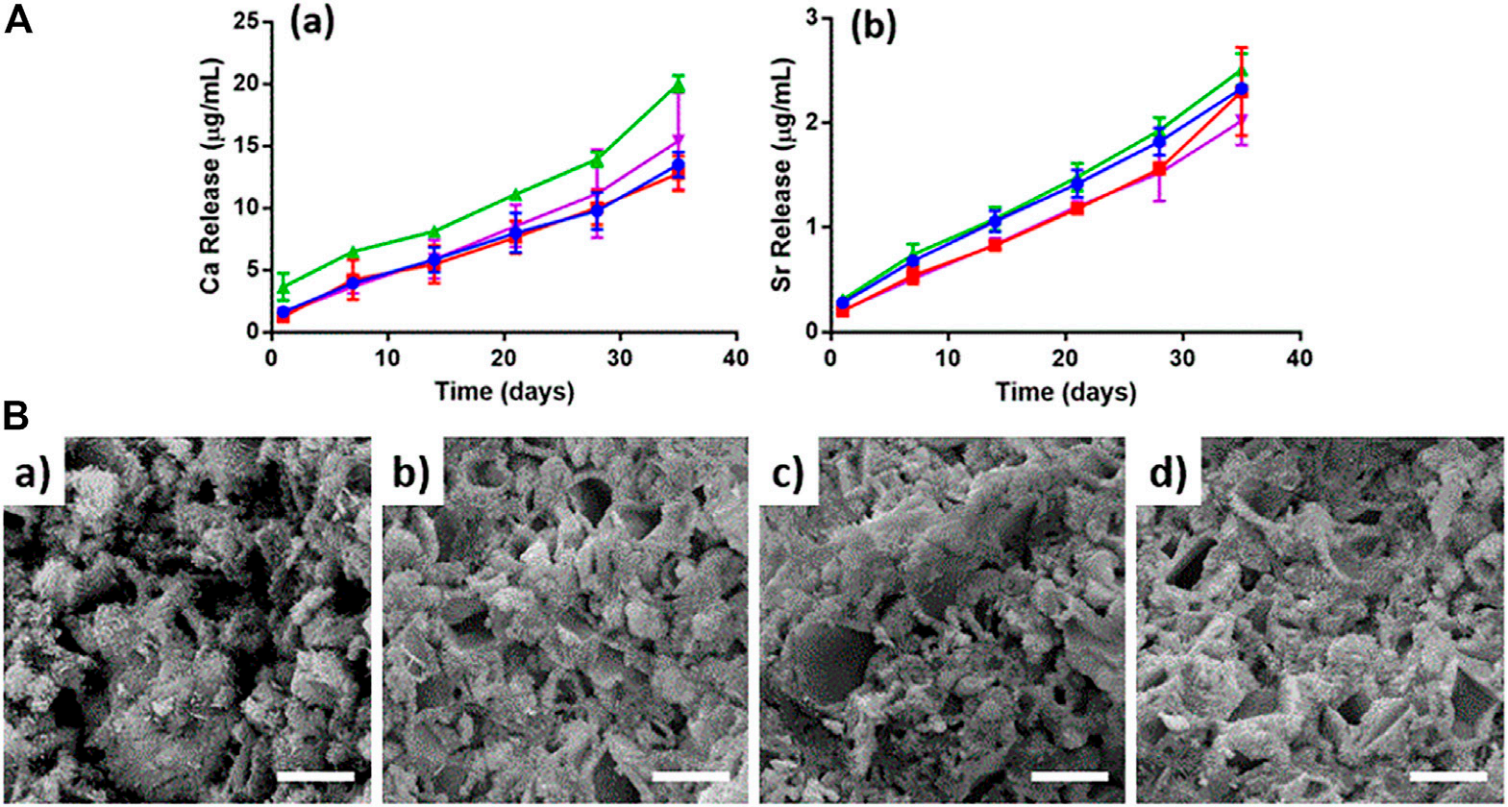
**(A)** Ion release up to 35 days: **(a)** Calcium, **(b)** Strontium. SrCPC (blue), SrCPC_NP (red), SrCPC_TC (green), SrCPC_NP-TC (violet); **(B)** SEM micrographs of cements: CPC **(a)**, CPC_TC **(b)**, CPC_NP **(c)**, CPC_NP-TC **(d)**. Scale bar = 5 µm.

Then, this scenario is complicated by the addition of NP-TC, leading to a significant slackening in TC release, possibly due to a combination of SrCPC matrix dissolution and TC desorption from NPs surface. Degradation tests of the bone cements were also performed up to 35 days, in terms of calcium and strontium ions release, exhibiting higher calcium release for the TC-containing formulations (Figure 5A). The microstructure of cements was also investigated by SEM, exhibiting flaky to needle-like HA crystals, particularly after addition of NPs (Figure 5B and Supplementary Figure S2).

The compressive strength of cement formulations was also evaluated at 7 days after soaking in HEPES at 37°C, exhibiting significant decrease only for the TC-containing formulations (Table 2).

**TABLE 2:** Compression strength of cements with and without NPs or TC, at 7 days after soaking in HEPES at 37°C.

	SrCPC	SrCPC_NP	SrCPC_TC	SrCPC_NP-TC
Compressive strength (MPa)	13.0 ± 4.4	9.4 ± 0.8	7.5 ± 1.4	6.3 ± 0.5

We observe that the addition of NPs, free TC and TC-functionalized NPs induces a reduction of compressive strength. On one side, the presence of NPs may also represent an obstacle hindering the grain interlocking phenomenon, typical of hardened CPC cements. On the other hand, the effect of the drugs on the mechanical performance of CPC was reported as difficult to predict, due to the possible chemical interaction of foreign molecules with the setting reaction (Ginebra et al., 2012). Notwithstanding, the cement exhibits mechanical strength suitable for application addressing bone regeneration in non load-bearing bone parts (Hernandez et al., 2001; Keaveny et al., 2001).

### 3.5 *In vitro* microbiological evaluation of bone cements

Antimicrobial tests were carried out on antibiotic containing cements (SrCPC_TC and SrCPC_NP-TC) using TC-free SrCPC_NP and SrCPC formulations as control samples. The following experiments were performed to evaluate the antibacterial properties of bone cement scaffolds either in planktonic or in biofilm conditions.

#### 3.5.1 Effect of bone cements scaffolds on bacterial planktonic cultures viability

**FIGURE 6:**
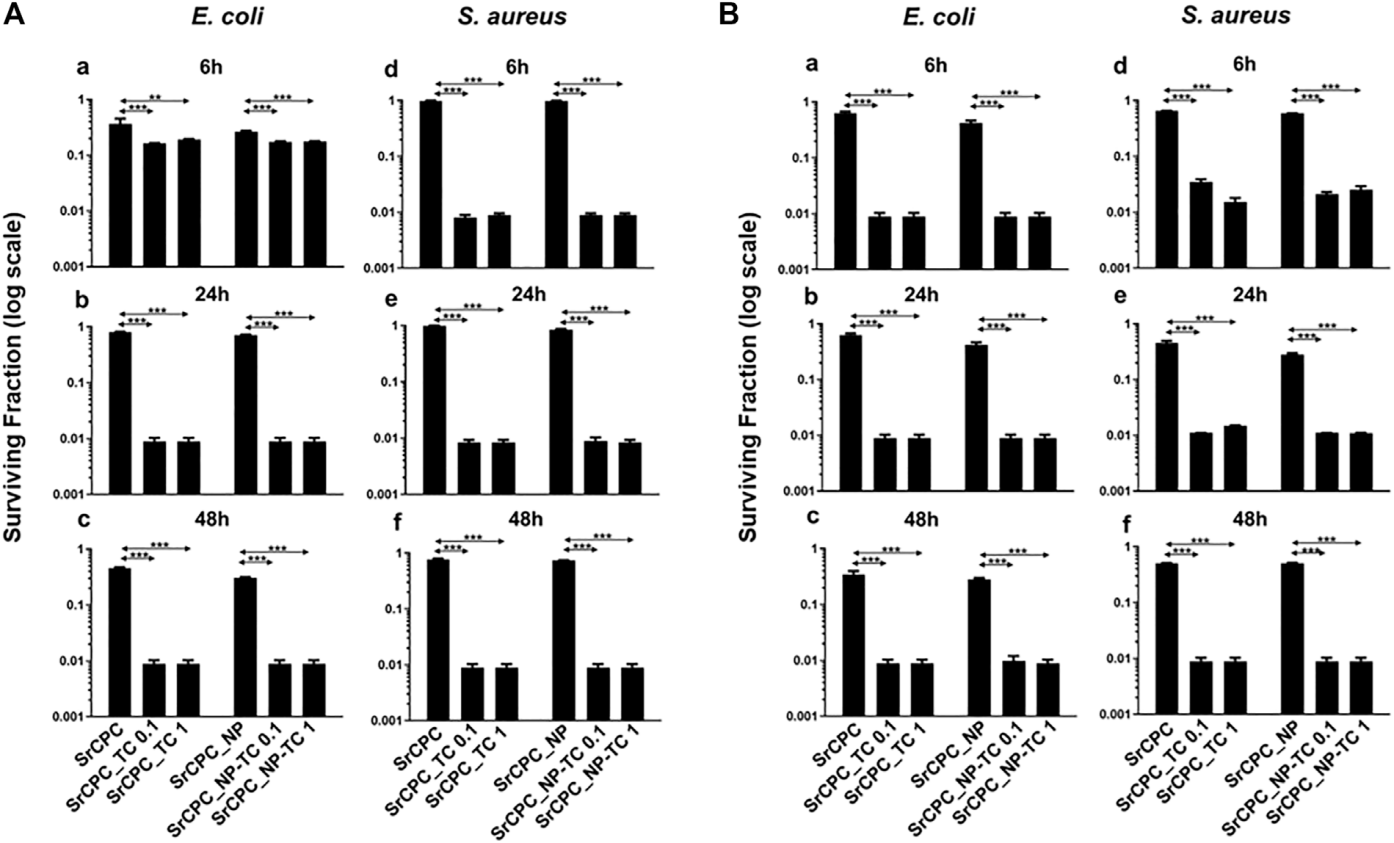
Bacterial viability of planktonic cultures **(A)** and adherent bacteria **(B)** to scaffold. *E. coli* **(a–c)** and *S. aureus* **(d–f)** were incubated through direct contact with scaffolds at 37°C for 6 h **(a–d)**, 24 h **(b–e)** and 48 h **(c–f)**, respectively. **(A)** The viability of planktonic bacteria was evaluated by MTT assay as described in the materials and methods section; **(B)** After the removal of planktonic bacteria at each time point, the viability of attached cells was determined by MTT assay. The data (log scale) were represented as the surviving fraction which is expressed as the ratio of the number of viable bacteria incubated with each scaffold over the number of bacteria grown in LB medium (Ctrl). Bars indicate mean values SD of the mean of results from two experiments. Statistical significance between the doped apatitic bone cements of the same group and their relative undoped samples are indicated as: **p < 0.01, ***p < 0.001. Statistical significance values detected for all samples related to bacteria grown in tissue culture plate with LB medium and no scaffolds: *p* < 0.05 (data not shown).

**FIGURE 7:**
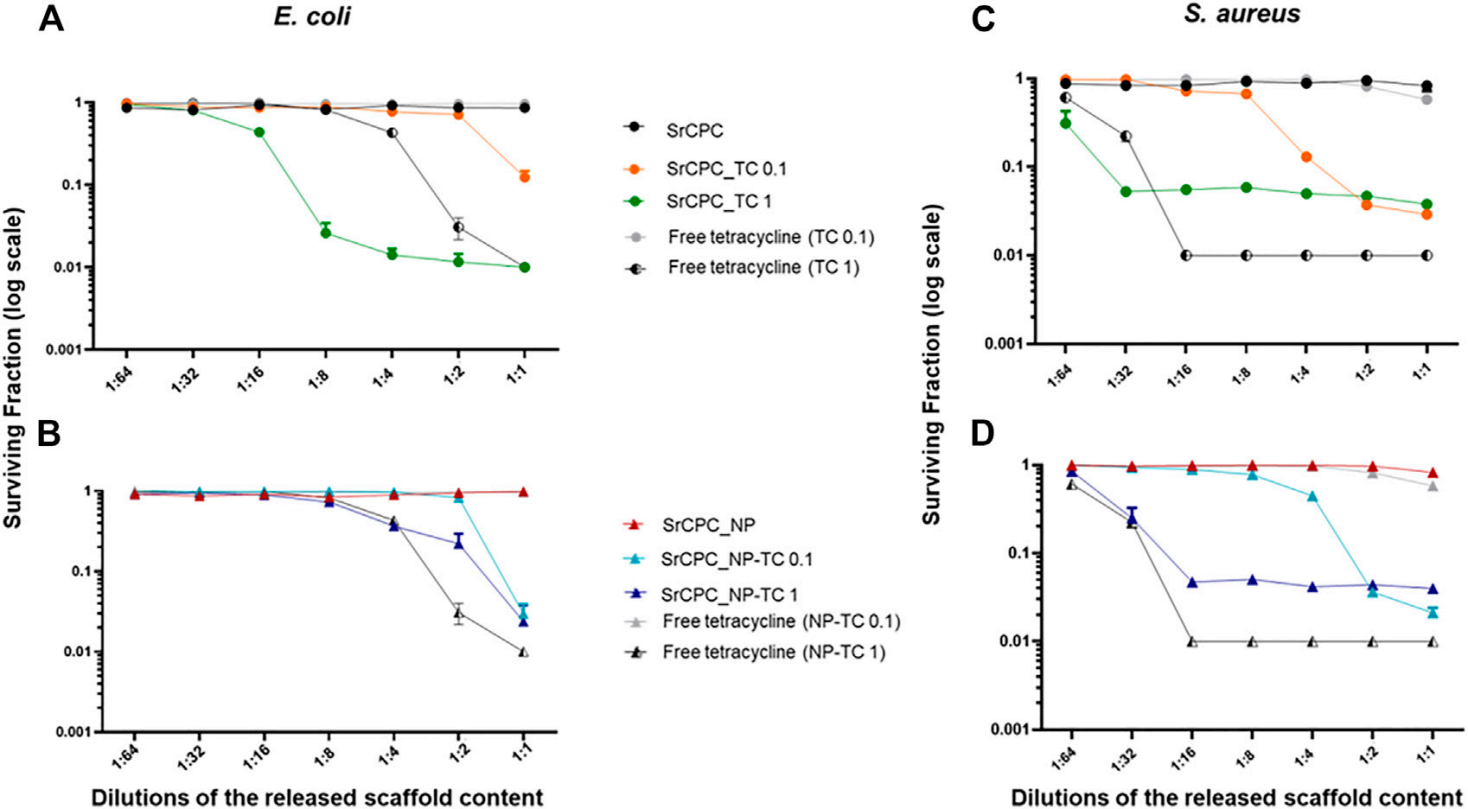
Bacterial viability of planktonic cultures by indirect contact. Each SrCPC_TC **(A**–**C)** and SrCPC_NP-TC **(B**–**D)** scaffolds, with their respective undoped samples (SrCPC, SrCPC_NP), were incubated with LB medium, for 24 h at 37°C. Tetracycline-HCl powder (indicated as free tetracycline 0.1; 1) was dissolved in LB according to the same concentrations released from SrCPC_TC and SrCPC_NP-TC scaffolds after 24 h at 37°C. The overnight solutions containing ions and antibiotics were diluted and incubated with planktonic *E. coli* **(A,B)** and *S. aureus* **(C,D)** cells for 24 h at 37°C, respectively. The data (log scale) were represented as the surviving fraction in respect to the control which is represented by bacteria grown in LB medium: *p* ≤ 0.05 (data not shown).

Bacterial viability was evaluated through the MTT colorimetric assay on planktonic cultures through direct contact (Figure 6) and indirect contact experimental set up (Figure 7). The direct contact tests were performed on at 6 h, 24 h and 48 h either on the supernatant, containing the planktonic bacteria (**Analysis 1**, Figure 6A) and on the bacteria adherent on the scaffold surface (**Analysis 2**, Figure 6B).

Both analyses of the direct test were important to be performed: the **analysis 1** allowed to determine whether the substances released from the scaffolds could contribute to reduce bacterial viability of both bacterial strains; the **analysis 2**, being performed on adherent bacteria to the scaffolds, could indicate whether the surface itself holds anti-adhesive properties. Finally, by indirect tests it was evaluated only the contribution of the released substances from the scaffolds on the bacterial viability.

Regarding the direct contact test, the analyses performed on the bacterial supernatant removed from the scaffolds after time-dependent incubation **(analysis 1)** (Figure 6A) revealed that both types of cements, SrCPC_TC and SrCPC_NP-TC, loaded with two different concentrations of TC (0.1 mg/ml and 1 mg/ml) showed a two logs reduction of the viability of both Gram-positive and Gram-negative bacteria after 6 h (Figure 6A,b–f) in comparison to their controls (SrCPC and SrCPC_NP). Significantly different effects were also exerted by both SrCPC_TC and SrCPC_NP-TC on *E. coli* and *S. aureus* cells survival at 6 h (Figure 6A). TC is an antibiotic that inhibits bacterial protein synthesis by preventing the association of aminoacyl-tRNA with bacterial ribosome. Therefore, to interact with the target, the antibiotic needs to cross the one or more membranes depending on bacteria (Chopra and Roberts, 2001). Gram-positive bacteria are characterized by the presence of a thick peptidoglycan layer associated to an inner cytoplasmic membrane; furthermore, the cell wall contains teichoic acids and lipoteichoic acids that are polysaccharides covalently attached to the peptidoglycan and inserted into the cytoplasmic membrane, respectively. Conversely, Gram-negative bacteria contain both a cytoplasmic and an outer membrane with a lipopolysaccharides (LPS), while a thin peptidoglycan layer is placed between the two membranes (Auer and Weibel, 2017). TC was reported to be less effective against Gram-negative than Gram-positive bacteria because of the presence of a second (outer) membrane (Epand et al., 2016; Rusu and Buta, 2021). In Gram-negative bacteria, TC indeed crosses the outer membrane through a cationic complex with Mg^2+^, using specific porins (OmpF and OmpC). Later, the antibiotic is attracted towards cytoplasm across the outer membrane by Donnan membrane potential, causing the accumulation of TC-Mg^2+^ complex in the periplasmic space where the antibiotic molecules dissociate from Mg^2+^ (Chopra and Roberts, 2001; Rusu and Buta, 2021). Since the molecules at this stage are uncharged and lipophilic, they can diffuse through the inner membrane and accumulate into the cytoplasm. In Gram-positive instead, the uptake of tetracycline across the cytoplasmic membrane is energy dependent and driven by the ΔpH component of the proton motive force. Within the cytoplasm, tetracycline molecules are likely to become chelated since the internal pH and divalent metal ion concentrations are higher than those outside the cell (Chopra and Roberts, 2001). Indeed, it is probable that the active drug species which binds to the ribosome is a Mg^2+^-tetracycline complex. Association of TC with the ribosome is reversible (at low concentration), providing an explanation of the bacteriostatic effects of these antibiotics. However, at high concentration this antibiotic can be a bactericidal agent (Rusu and Buta, 2021).

The results of **analysis** 2 of the direct contact test related to the *E. coli* and *S. aureus* adhesion onto the scaffolds surfaces are reported in Figure 6B. Both the antibiotic containing scaffolds (SrCPC_TC and SrCPC_NP-TC) retained antibacterial properties at 6 h of incubation, showing two logs of viability reduction. The anti-adhesive ability of bone cements can be ascribed to the sustained release of TC, associated to the release of Ca^2+^, PO_4_^3-^ and Sr^2+^ ions showing relevant antibacterial effects, as previously observed (Sampath Kumar et al., 2015; Baheiraei et al., 2021; Jiao et al., 2021). Moreover, on SrCPC and SrCPC_NP the adhesion was reduced for *E. coli* after 48 h (Figure 6B,c) and for *S. aureus* after 24 h (Figure 6B,e–f). The reduced adhesion was probably due to the characteristics of the surface because it is known in literature that properties such as surface charge density and roughness affect bacterial adhesion (Zheng et al., 2021). Surface charge involves van der Waals force and electrostatic interactions that are the major forces in bacterial adhesion onto material surfaces. Considering that bacteria are usually negatively charged, due to carboxyl groups, amino and phosphate groups on their cell wall, more adhesion is often observed on positively charged surfaces. Another property that can influence adhesion is surface roughness. Higher is the degree of roughness, more reduced will result bacterial adhesion, because of the decreased contact area between bacteria and surface (Zheng et al., 2021) as well as hydroxyapatite (Maleki-Ghaleh et al., 2021). In Figure 6 was reported the statistical analysis of TC loaded cements in comparison to their respective control. The statistics between SrCPC control compared to SrCPC_NP-TC cements and SrCPC-NP compared to SrCPC-TC was reported in Supplementary Table S2 for both planktonic (A-B) and adherent bacteria (C-D). The analysis showed a significant difference of TC loaded cements (*p* < 0.05). Moreover, it was observed a significant difference between SrCPC and SrCPC_NP controls after 24 h and 48 h of incubation with both planktonic bacteria (Supplementary Tables S2A–B) and after 6 h and 24 h of both bacterial adhesions (Supplementary Tables S2C–D).

The antibiotic solutions released from SrCPC_TC 1 and SrCPC_NP-TC 1 samples were tested to evaluate, as indirect contact, the viability of both bacterial strains in comparison to free TC concentrations. As expected, the solution released from both types of apatitic bone cement scaffolds showed an antibacterial effect towards both Gram-negative and positive bacteria (Figure 7).

In particular, the solution released from SrCPC_TC 1 showed a viability reduction of two logs against *E. coli* and this was more efficient in comparison to the free added tetracycline (TC 1) at the same concentration (Figure 7a). Conversely, the SrCPC_NP-TC 1 solutions were not as effective (ca. One log of reduction) as the free TC 1 (Figure 7b). No significant differences were detected against *S. aureus* by using SrCPC_TC and SrCPC_NP-TC (Figures 7c,d). In general, a reduction in viability of ca. One log for both samples was observed if compared to free TC (2 logs of reduction).

#### 3.5.2 Effect of bone cements scaffolds on biofilm formations

**FIGURE 8:**
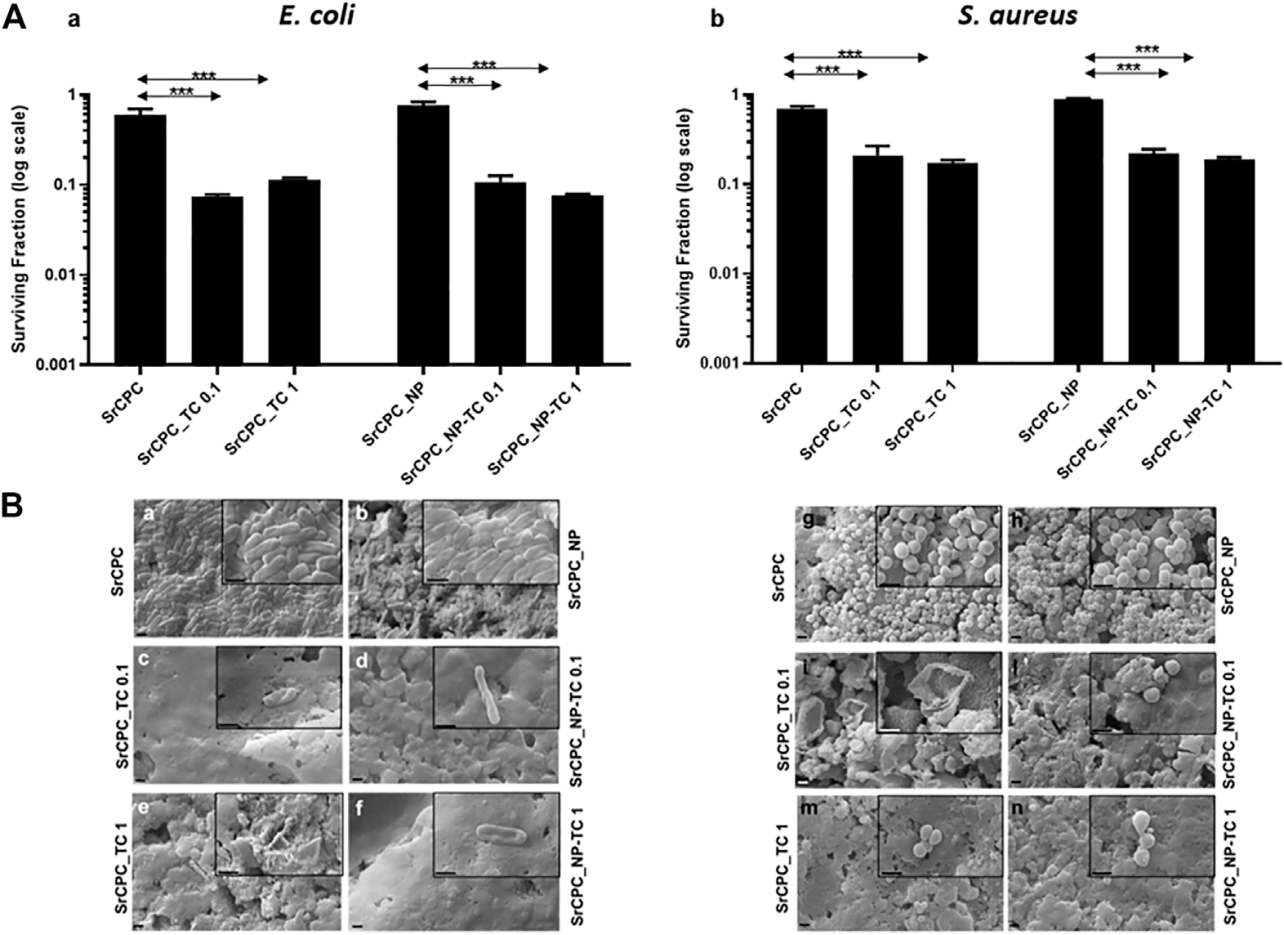
Effect of the tested scaffolds on the formation of bacterial biofilms (Pre-biofilm conditions). **(A)** Viability of bacterial biofilms. *E. coli* **(A,a)** and *S. aureus* **(A,b)** were incubated for 24 h at 37°C on different scaffolds in order to allow the formation of biofilm as indicated in the materials and methods section. After overnight incubation, the planktonic bacteria were removed, and the cell viability of bacterial biofilm formed on the scaffolds were tested with MTT. The control is represented by biofilm grown on tissue culture plate in LB medium as indicated in materials and methods section. The data (log scale) were represented as the surviving fraction. Bars indicate mean values SD of the mean of results from two experiments. Statistical significance between the doped apatitic bone cements of the same group and their relative undoped samples are indicated as: ***p < 0.001. Statistical significance values detected for all samples related to bacteria grown in tissue culture plate with LB medium and no scaffolds: *p* < 0.05 (data not shown); **(B)** SEM images of biofilm. *E. coli* **(Ba–f)** and *S. aureus* **(Bg–n)** biofilms were observed with SEM. Images at magnification 15KX (1 μm bar) and 50KX (insets, 1 μm bar), respectively. SrCPC **(a,g)**; SrCPC_NP **(b,h)**; SrCPC_TC 0.1 mg/ml **(c,i)** and 1 mg/ml **(e,m)**; SrCPC_NP-TC 0.1 mg/ml **(d,l)** and 1 mg/ml **(f,n)**.

**FIGURE 9:**
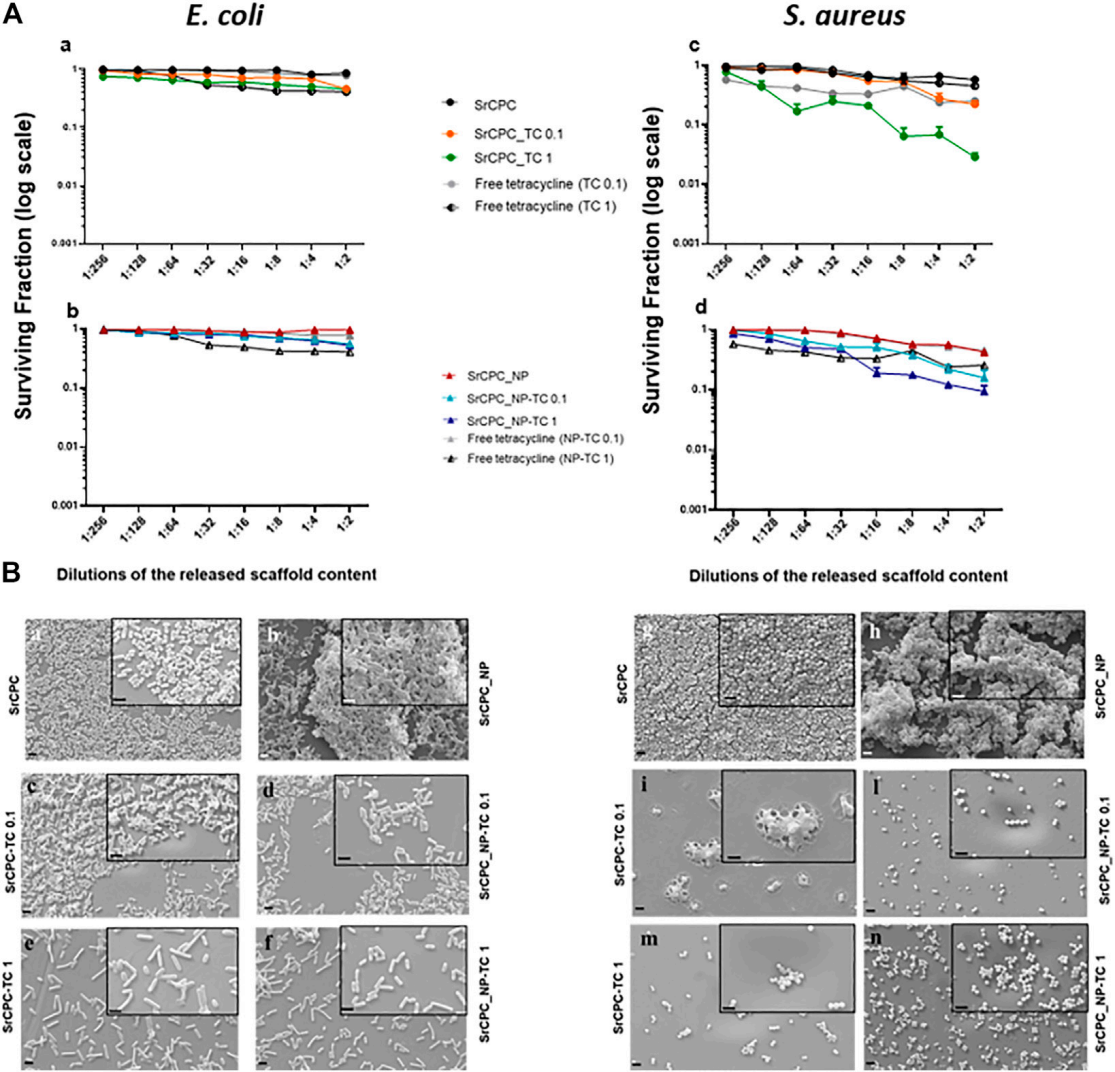
Effect of scaffold released contents on bacterial preformed biofilms (Post-biofilm conditions). **(A)** Viability of bacterial biofilms. Each scaffold was incubated with LB medium, for 24 h at 37°C. The overnight solutions containing ions and tetracycline were added at the overnight preformed bacterial biofilms of *E. coli* **(a,b)** and *S. aureus* **(c,d)** at the dilutions indicated for 24 h at 37°C. Tetracycline-HCl powder (in figure indicated as free tetracycline 0.1; 1) was dissolved in LB according to the same concentrations released from SrCPC_TC and SrCPC_NP-TC scaffolds after 24 h at 37°C. The data (log scale) were represented as the surviving fraction. *p* < 0.05 (data not shown). **(B)** SEM images. Bacteria were seeded onto coverslips and incubated for 24 h at 37°C for biofilm formation. After the removal of planktonic bacteria form the coverslips, the previously obtained overnight solutions form the doped and undoped scaffolds were serially diluted and incubated for 24 h at 37°C onto each biofilm. At the end of incubation after removal of the medium, the cells were treated as indicated in materials and methods and observed by SEM. Images of the samples were performed at magnification 6KX (2 μm bar) and 15KX (insets, 1 μm bar), respectively. SrCPC **(a,g)**; SrCPC_NP **(b,h)**; SrCPC_TC 0.1 mg/ml **(c,i)** and 1 mg/ml **(e,m)**; SrCPC_NP-TC 0.1 mg/ml **(d,l)** and 1 mg/ml **(f,n)**.

Given the promising results from planktonic and adherent bacteria, the ability of SrCPC scaffolds in preventing the formation of bacterial biofilms (***pre-biofilm conditions***) (Figure 8) or reducing the preformed biofilms (***post-biofilm conditions***) (Figure 9) was also investigated, as also previously reported (Balato et al., 2019; Cara et al., 2022; Jacquart et al., 2022).

In ***pre-biofilm conditions,*** the formation of both biofilms is prevented by SrCPC_TC and SrCPC_NP-TC at both concentrations when bacterial cells are directly seeded on the bone cement scaffolds and allowed to grow for 24 h at 37°C. SrCPC_TC and SrCPC_NP-TC scaffolds showed, in *E. coli*, one log of reduction in respect to their sample controls (Figure 8Aa). *S. aureus* biofilm formation was, instead, reduced of about half log from SrCPC_TC and SrCPC_NP-TC (Figures 8A,b). The statistical analysis of cements between bacterial biofilms was represented in Supplementary Table S3. The analysis showed a significant difference between *S. aureus* TC loaded cements compared to *E. coli* cements control (*p* < 0.05). In addition, SrCPC_NP and SrCPC_TC 0.1 cements were statistically significant in both bacterial biofilms (*p* < 0.05).

This minor susceptibility of *S. aureus* biofilm to tetracycline-containing bone cements, with respect to *E. coli* biofilm, could be explained by the resistance mechanisms that bacteria show against TC, as previously described. Moreover, the results on sample controls (SrCPC and SrCPC_NP) obtained from assessing the viability of biofilm in pre-biofilm conditions showed a small reduction of viability for both bacteria. These data demonstrated that the adhesion on bone cements was slightly hindered from scaffold surfaces, we attributed such an effect to surface nano-roughness. Although this property may contribute to decrease bacterial adhesion (Zheng et al., 2021), the most anti-adhesive effect of SrCPC bone cements was due to the presence of tetracycline that reduced both biofilms formation of ca. One log.

Data were supported by SEM observations showing *E. coli* and *S. aureus* biofilms formations on SrCPC and SrCPC_NP (Figure 8B); further study should be performed to measure their thickness. On the contrary, SrCPC_TC (Figures 8B,c,e,i,m) and SrCPC_NP-TC (Figures 8Bd,f,l,n) scaffolds, did not allow the formation of biofilms.

In ***post-biofilm conditions***, TC and ions released from the scaffolds after 24 h incubation at 37°C were tested in a dose-dependent manner against both bacterial preformed biofilms (Figure 9A). Free tetracycline was used as a positive control at the same concentrations released from the scaffolds. As illustrated in Figure 9A the solutions, particularly those incubated either with SrCPC_TC 1 or SrCPC_NP-TC 1, were more effective against *S. aureus* biofilms **(** Figures 9Ac,d) in comparison to *E. coli* (Figures 9Aa,b). The efficacy of tetracycline on Gram-positive bacteria (Rusu and Buta, 2021) is due to the different mechanism of diffusion, related to the different structure of cell wall between Gram-positive and negative bacteria (Auer and Weibel, 2017). These viability data are also supported by SEM images (Figure 9B).

Overall, our results showed that Sr-doped apatitic bone cements are capable of effective reduction of *E. coli* and *S. aureus* as evaluated in planktonic and biofilm conditions. Particularly, we showed that the release of TC, associated to the intrinsic physico-chemical features of bone cement, such as the ability of multiple Ca^2+^, PO_4_^3-^ and Sr^2+^ ion release as well as the surface nanotexture can play a relevant role in limiting bacterial adhesion, viability, and formation of bacterial biofilms. We established the conditions to load different amounts of TC on HA-NPs, that were incorporated and distributed throughout the whole volume of the hardened SrCPC cements, thus obtaining ability to modulate the TC release process and to achieve a release profile sustained along weeks. Devices with regenerative properties associated to sustained long-term antibacterial effects, effective also in preventing biofilm formation, may have great clinical relevance thus overcoming the well-known problems related to systemic administration such as reduced drug availability at the target site, adverse side effects and promising also to overcome the bacterial resistance to antibiotics. In respect to the ability of sustained drug delivery, the cements TC release process was modulated by a combination of factors: 1) desorption from NPs surface modulated by dissolution processes involving the cement and the NPs themselves; 2) diffusion of TC through the nano/micro-pores network of the SrCPCs. Such a canaliculi network, spontaneously formed during the reprecipitation process yielding the cement hardening, was already shown to play a key role for new bone penetration and osteointegration during a previous *in vivo* test in rabbit femur defects (Sprio et al., 2016). The microbiological results obtained in the present work show that such feature, together with a composition capable to release bioactive Ca^2+^, PO_4_^3-^ and Sr^2+^ ions and with a diffuse nanotexture providing high specific surface area, are all key aspects making apatitic bone cements promising as multifunctional devices. Indeed, nano-crystallinity associated with ion-release ability were previously shown as relevant factors to exhibit osteogenic and antibacterial properties in apatitic nanoparticles (Ballardini et al., 2018; Sprio et al., 2020). In this paper we show that such features are effective also when dealing with bone cements developed as 3D bodies, characterized by mechanical properties suitable to regenerate non load-bearing bony defects, typical for instance in the case of tumoral bone resections. Indeed, the chemical similarity of TC with anthracycline (i.e., doxorubicin) indicates that our approach could be carried out also with anticancer drugs and potentially with a large number of bioactive molecules, thus representing a promising solution to sustain bone regeneration also in case of bone cancer or other co-morbidities.

## 4 Conclusion

In the present work, injectable self-hardening CPC bone substitutes partially substituted with Sr^2+^ ions and capable of sustained release of TC were developed. The modulation of the drug release profile was achieved by linking TC with HA-NPs appositely synthesized and mixed with the cement precursor before mixing with liquid components. This study was carried out to evaluate the potential capacity to contrast post-operative infectious complications by osteogenic and nanostructured porous bone CPCs. Extensive *in vitro* microbiological characterization demonstrated the intrinsic ability of the Sr-doped bone cement to contrast infections caused by planktonic *E. coli* and *S. aureus* bacteria. In addition, the TC-containing cements showed the ability to contrast the formation of biofilms suggesting penetration and effective action of the antibiotic and released bioactive ions into the biofilms. Effective antibacterial character even in absence of antibiotic drugs was also shown, thus suggesting intrinsic compositional and textural features of the bone cement such as surface nano-roughness, possibly acting as intrinsic factors capable to hinder biofilm adhesion and organization. These results give a promising perspective regarding the reduction or a more effective use of antibiotic drugs, thus aiding to circumvent bacterial resistance phenomena, which are among the major threats in medicine, particularly in orthopedics.

## Data Availability

The raw data supporting the conclusions of this article will be made available by the authors, without undue reservation.
