# Supplementary material for: Strontium-doped apatitic bone cements with tunable antibacterial and antibiofilm ability
Source: Front Bioeng Biotechnol. 2022 Dec 9;10:969641. doi: 10.3389/fbioe.2022.969641 (PMC9780487; doi:10.3389/fbioe.2022.969641)

**_Supplementary Information_**

**_Strontium-doped apatitic bone cements with tunable antibacterial and antibiofilm ability_**

**Massimiliano Dapporto^1^, Marta Tavoni^1^, Elisa Restivo^2^, Francesca Carella^1^, Giovanna Bruni^3^, Laura Mercatali^4^, Livia Visai^1,2,5^, Anna Tampieri^1^, Michele Iafisco^1*^, Simone Sprio^1*^**

^1^Institute of Science and Technology for Ceramics, National Research Council of Italy, Faenza, Via Granarolo 64, Italy

^2^Molecular Medicine Department, Center for Health Technologies, UdR INSTM, University of Pavia, Pavia, Italy

^3^University of Pavia, Department of Chemistry, Physical Chemistry Section; Center for Colloid and Surfaces Science, Pavia, Italy

^4^IRCCS Istituto Romagnolo per lo Studio dei Tumori (IRST) "Dino Amadori", Osteoncology and Rare Tumors Center, Meldola, Italy

^5^Medicina Clinica-Specialistica, UOR5 Laboratorio di Nanotecnologie, ICS Maugeri. IRCCS, Pavia, Italy

*** Correspondence:**Michele Iafisco
[michele.iafisco@istec.cnr.it](mailto:michele.iafisco@istec.cnr.it)

Simone Sprio

[simone.sprio@istec.cnr.it](mailto:simone.sprio@istec.cnr.it)

**
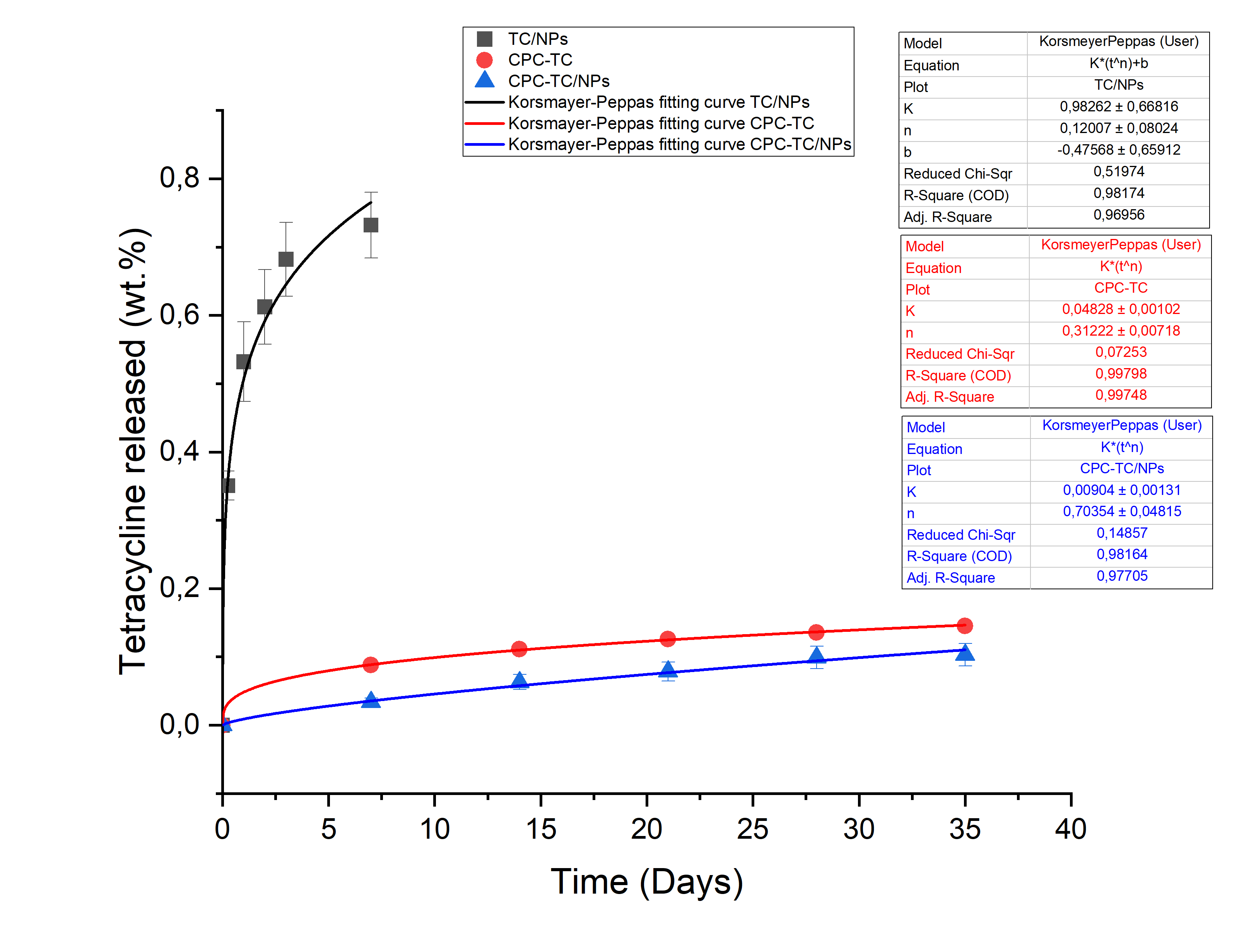
**

Suppl. Figure 1 – Kinetic release profiles of tetracycline from NP-TC (black-line), SrCPC-TC (red-line) and SrCPC-NP_TC (blue-line) with respective fitting curves

Suppl. Table I. Interpretation of the Korsmeyer-Peppas exponent for cylindrical samples.

| *n* exponent value | Release regime | Release kinetic and mechanisms |
| --- | --- | --- |
| 0 < n < 0,45 | Hindered Fickian diffusion | Diffusive regime with hampered release. |
| n = 0,45 | Fickian diffusion (case I) | First-order kinetic where diffusion is the main release mechanism |
| 0,45 < n < 1 | Anomalous transport | Diffusion, associated to other mechanisms (e.g. matrix erosion or swelling) |
| n = 1 | Non-Fickian transport (case II) | zeroth order kinetic and is typical of kinetics governing by phenomena of polymer degradation and relaxation or degradation/dissolution of monolithic system |
| n > 1 | Super case II | Extreme form of transport that usually occurs when severe modification in the matrix take place |


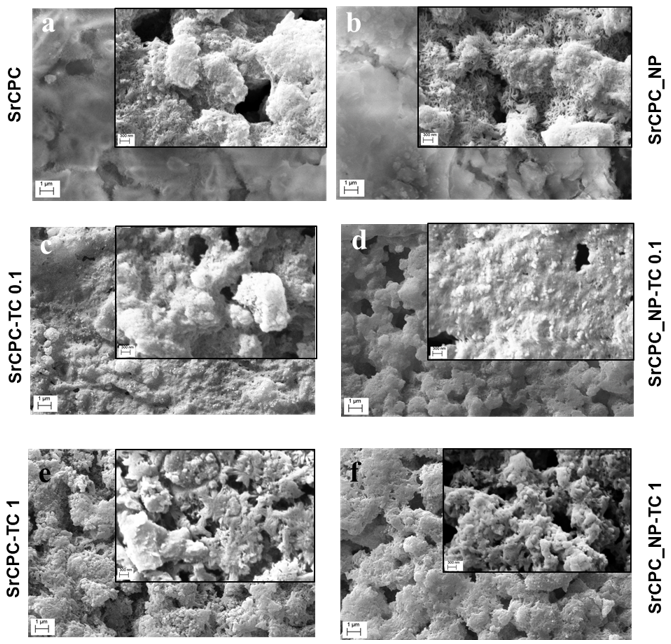


**Suppl. Figure 2 - SEM images of the scaffolds.** SEM images of the scaffolds were performed at magnification 15KX (1 µm bar) and 50KX (insets, 300 nm bar), respectively. (a) SrCPC; (b) SrCPC_NP; (c) SrCPC_TC 0.1; (d) SrCPC_NP-TC 0.1; (e) SrCPC_TC 1; (f) SrCPC_NP-TC 1.

**Suppl. Table II - Statistics of planktonic culture viability (A-B) and bacterial adhesion (C-D).** ANOVA followed by Bonferroni’s test was performed to compare SrCPC with SrCPC_NP scaffolds incubated through direct contact for 6h, 24h and 48h with *E. coli* (**A, C**) and *S. aureus* (**B, D**). *p < 0.05, **p < 0.01 and ***p < 0.001. ns = not significant value.


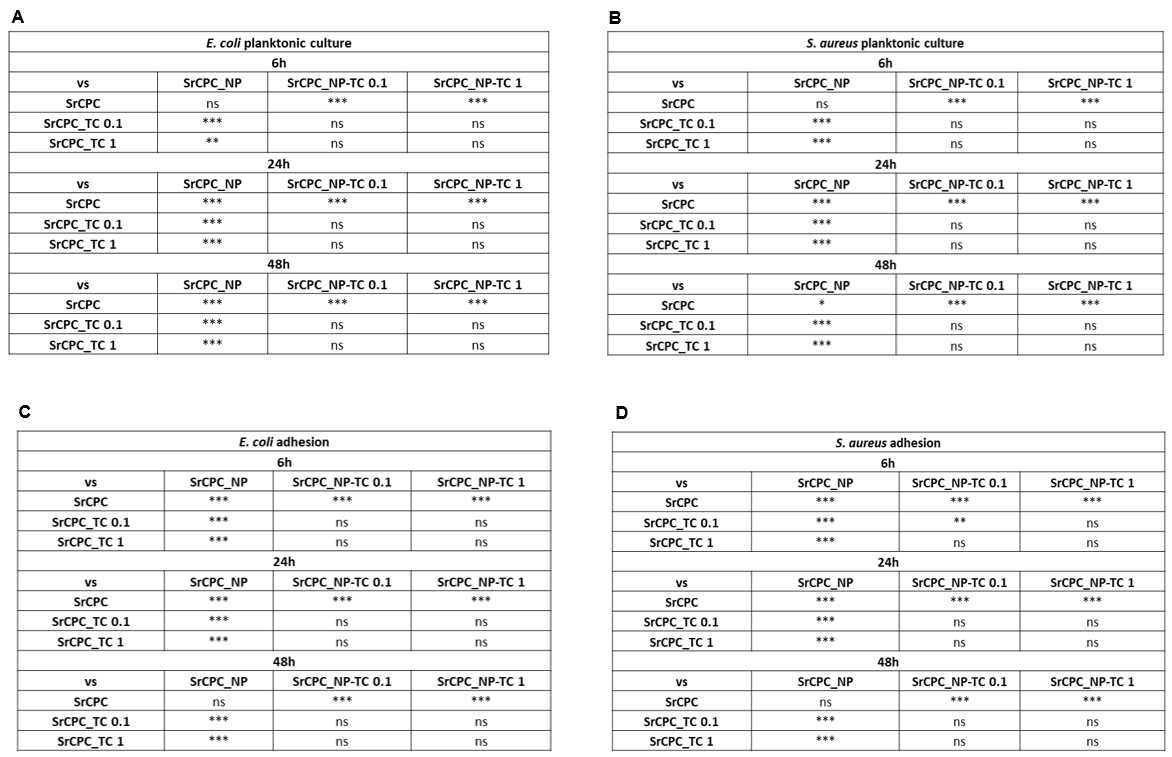


**Suppl. Table III - Comparison between bacterial biofilms.** ANOVA followed by Bonferroni’s test was performed to compare the viability of *E. coli* and *S. aureus* biofilms formed on scaffolds (pre-biofilm conditions). *p < 0.05, **p < 0.01 and ***p < 0.001. ns = not significant value.


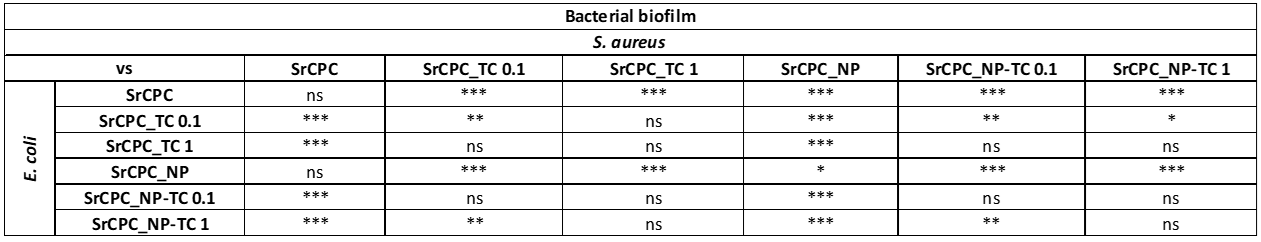

Supplement: Supplementary file 1 [file DataSheet1.DOCX]
